# Downregulation of TRPC4 and TRPC5 Inhibits Smooth Muscle Cell Proliferation without Affecting Endothelial Cell Proliferation

**DOI:** 10.1155/2021/2949986

**Published:** 2021-11-27

**Authors:** Wenjun Zeng, Yinan Ji, Haiping Zhang, Liusheng Chen, Li Du, Ruiwei Guo

**Affiliations:** ^1^Department of Cardiology, Kunming Medical University, The 920th Hospital, Kunming, Yunnan 650032, China; ^2^The 75th Group Army Hospital of PLA, Kunming, Yunnan 671000, China; ^3^Department of Cardiology, 920th Hospital of Joint Logistics Support Force, PLA, Kunming, Yunnan 650032, China

## Abstract

**Aims:**

The main treatment for coronary heart disease is percutaneous coronary intervention (PCI), and drug-eluting stents are designed to inhibit vascular smooth muscle cell (VSMCs) proliferation and migration causing restenosis by releasing pharmacological agents into the vessel wall. Once drug-eluting stents are deployed, these pharmacological agents exert many biological effects in the coronary circulation, not only inhibition of VSMCs but also extension to vascular endothelial cells (VECs). The purpose of this study was to explore target molecules that inhibit VSMCs proliferation without affecting VECs.

**Methods:**

mRNA and protein expressions of transient receptor potential channels (TRPCs) in cultured VSMCs and VECs were determined by western blotting and RT-qPCR. VSMCs and VECs proliferation was evaluated using CCK-8 assays and western blotting of proliferating cell nuclear antigen (PCNA). Calcium backfilling assays were performed to detect intracellular calcium ion concentration in cultured VSMCs and VECs.

**Results:**

The TRPC6 expression was more abundant in VECs than VSMCs, while TRPC4 and TRPC5 expressions were more abundant in VSMCs than VECs. Knockdown of TRPC4 or TRPC5 alone had no remarkable inhibitory effect on VSMC proliferation. Synergistic knockdown of TRPC4 and TRPC5 inhibited the proliferation of VSMCs, declined the expression of the PCNA, and reduced the intracellular calcium ion concentration but not VECs.

**Conclusion:**

These data suggest that concurrent inhibition of TRPC4 and TRPC5 inhibits VSMCs proliferation without affecting VECs, thus providing novel targets for developing pharmacological agents for drug-eluting stents.

## 1. Introduction

In recent years, coronary heart disease has become one of the major diseases endangering human health, and percutaneous coronary intervention (PCI) remains the main treatment [1]. Drug-eluting stents are the most widely used stents in clinical practice due to their inhibition on proliferation and migration of vascular smooth muscle cells (VSMCs) and prevention on restenosis by releasing pharmacological agents into the vessel wall [2, 3]. Once drug-eluting stents are deployed, these pharmacological agents exert many biological effects in the coronary circulation, not only inhibition of VSMCs but also extension to vascular endothelial cells (VECs). However, the delayed proliferation of VECs exposes the stent to the lumen of the blood vessel for a prolonged time, increasing the risk of thrombosis [4]. Normal VECs can maintain vascular homeostasis and inhibit thrombosis by regulating vascular tension, preventing thrombosis, and regulating inflammation. Excess apoptosis of VECs is a preliminary event in thrombosis development [5]. In order to prevent platelet aggregation induced by the stent metal framework and thrombus formation that blocks blood vessels, patients receiving PCI must receive dual antithrombotic therapy [6]. However, dual antithrombotic treatment after stenting increases the risk of bleeding complications in patients [4]. Identifying a drug target that can inhibit the proliferation of smooth muscle cells without affecting the proliferation of endothelial cells could greatly reduce these unwanted side effects in patients taking dual antiplatelet drugs after surgery and shorten the time required for dual antithrombotic therapy.

Ca^2+^ is a second messenger that plays an important role in cellular signal transduction and regulates numerous physiological activities such as proliferation, differentiation, transmission of information, and gene transcription [7]. Changes in intracellular free calcium concentration directly influence cell proliferation [8, 9]. There are various types of calcium channels, including voltage-dependent calcium channels (VDCs), receptor-operated calcium channels (ROCs), and store-operated calcium channels (SOCs). Among them, SOCs play a very important role in regulating the functions of cells, including calcium influx, cell migration, and cell proliferation [10].

Transient receptor potential channels (TRPCs) and ORAI are important components of SOCs. In HEK293 cells, lymphocytes, and fibroblasts, ORAI proteins enhance store-operated calcium entry [11]. If the calcium ion concentration in the endoplasmic reticulum calcium pool is reduced or depleted, STIM activation in the endoplasmic reticulum transmits information to TRPC and ORAI located on the cell membrane, and this mediates TRPC and ORAI. The opening of these two calcium-regulated protein channels causes extracellular calcium influx and ultimately regulates cell growth and proliferation [12].

The cation transporter Na^+^/Ca^2+^ exchange (NCX) is widely distributed on cell membranes, where it precisely and rapidly regulates the concentration of Ca^2+^ in the cytoplasm, which in turn affects many cell functions, such as signal transduction, cell growth and development, and the excitation-contraction coupling of excitable cells [13, 14]. Previous studies have shown that TRPC and NCX together regulate the proliferation of VSMCs [15], but it is not clear whether SOC and NCX influence both smooth muscle cells and endothelial cells. The study aimed to develop novel drug-eluting therapies that could reduce the time needed for treatment. To this end, we explored differences in the expression of TRPCs in VSMCs and VECs to identify target molecules that inhibited the proliferation of VSMCs without affecting the proliferation of VECs.

## 2. Materials and Methods

### 2.1. Cell Culture

Human coronary artery VSMCs and VECs (Bnbio, China) were cultured as described previously [16, 17] in 10% fetal bovine serum (FBS; Sciencell, San Diego, USA) + Dulbecco's Modified Eagle's Medium (DMEM; HyClone, USA) at 37°C in a 5% CO_2_ incubator for 8–10 passages.

### 2.2. Western Blotting

Cell lysates of cultured VSMCs and VECs were prepared, and the proteins were quantified by the bicinchoninic acid (BCA) method. After separated by 10% SDS-PAGE, the protein samples were then transferred to a PVDF membrane and blocked with 5% skim milk in TBST. The membrane reacted with anti-TRPC1, anti-TRPC4, anti-TRPC5, anti-TRPC3, anti-TRPC6, and anti-*β*-actin antibodies, with secondary antibody following standard western blotting procedures. Immunoblots were photographed by the use of the Bio-Rad image analysis system (Bio-Rad, Hercules, CA), and their densimetric analyses were performed using Quantity One v4.6.2 software. Anti-TRPC1, anti-TRPC4, and anti-TRPC5 antibodies were purchased from Novus Biologicals (Littleton, CO, USA). Anti-TRPC3, anti-TRPC6, anti-PCNA, and anti-*β*-actin antibodies were purchased from Cell Signaling Technology (USA).

### 2.3. RNA Isolation and Real-Time qPCR (RT-qPCR)

Total RNA was isolated with TRIzol reagent (Invitrogen, USA) according to the manufacturer's instructions. The PCR was directly monitored by a Bioer CFX96 real-time PCR sequence detection system. We used template RNA and primers to reverse-transcribe total RNA into cDNA after annealing at 25°C for 5 min, followed by extension at 42°C for 60 min and inactivation at 70°C for 15 min. The single-stranded cDNA was amplified by qPCR using 40 cycles. We searched the target gene sequence in the GenBank database and then used Primer 6.0 software to design primers with good specificity and a similar annealing temperature. To ensure the success of the experiment, two pairs of primers were designed for each gene, and the best pair was selected for subsequent experiments. Primer sequences are listed in Table 1.

### 2.4. Cell Transfection

VSMCs and VECs after passage 10 were transfected with siTRPC4 (5′-GGCCTAAATCAATTGTACT-3′) and siTRPC5 (5′-GACACGAATTCACCGAGTT-3′), both designed by RiboBio (China), using Lipofectamine 2000 reagent (Invitrogen). The transfection concentration of siRNAs was optimized according to the product specification. After 24 h of siRNA transfection, RNA was extracted from cells, cDNA was obtained by reverse transcription, and mRNA levels of TRPC4 and TRPC5 were measured by RT-qPCR.

### 2.5. CCK-8 Assays

Cells in the logarithmic growth phase were digested, cell suspensions were prepared, and cells were counted, inoculated into 96-well cell culture plates, and incubated for 2–4 h until adhered. Next, 200 *μ*L of fresh complete medium was replaced, CCK-8 reagent (Gibco) was added (20 *μ*L per well) every 24 h (at least three replicate wells per group), and cells were incubated and continually monitored for 5–7 days to plot curves. Cell counting was also performed.

### 2.6. Measurement of Intracellular Calcium Ion Concentration

For calcium experiments, cells were resuspended in phosphate-buffered saline (PBS), Fura-2AM was added to a final concentration of 5 *μ*mol/L, HBSS solution (containing 1.5 mmol/L CaCl_2_) was also added, and the cell density was adjusted to 1 × 10^6^/ml at a temperature of 37°C over 30 min in the dark. A fluorescence spectrophotometer was used for detection at excitation wavelengths of 340 nm and 380 nm scanned alternately, and the fluorescence intensity of the emitted light was measured. All traces were representative of several independent experiments.

### 2.7. Statistical Analysis

SPSS 17.0 software was used for statistical analysis. The results are expressed as mean ± standard error of the mean (SEM). Analysis of variance (ANOVA) was performed using Dunnett's *t*-tests for multiple comparisons, and *p* < 0.05 was considered statistically significant.

## 3. Results

### 3.1. Morphological Characteristics of VSMCs and VECs

Microscopy revealed that when the VSMCs' confluence reached 100%, cells displayed a very long fusiform shape, compared with the fusiform when the confluence reached 50% to 60%; the length of VSMCs increased significantly, and the cell arrangement was regular, with relatively clear cell boundaries. When cells grew to 100% confluence, VECs did not exhibit this typical fusiform structure and instead adopted a rhombic shape, the refractive index was slightly lower, and the cell outline was not clear. Morphological observation of VSMCs and VECs is detailed in Supplementary Materials 1.

### 3.2. Expression of TRPCs in VSMCs and VECs

**Figure 1:**
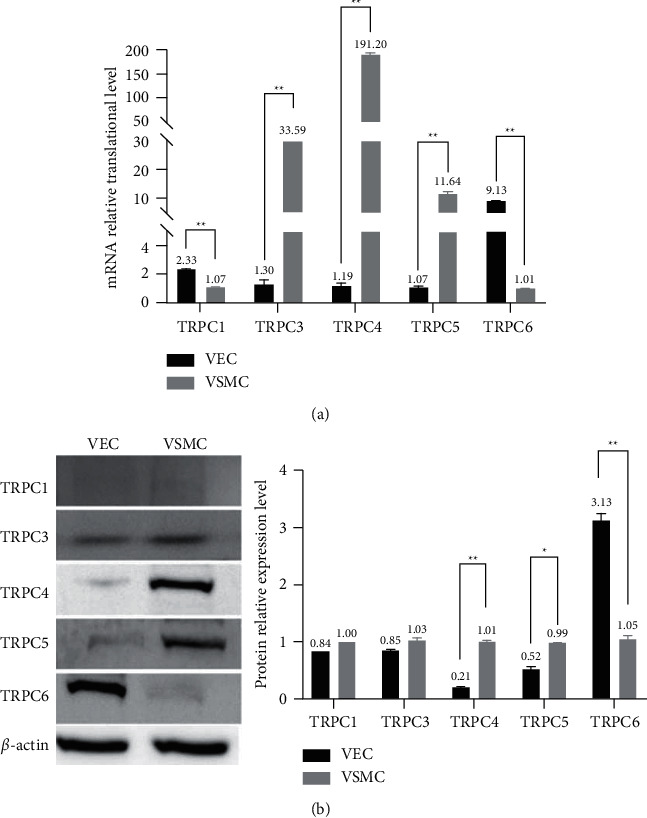
(a) mRNA expression levels of TRPCs (TRPC1, TRPC3, TRPC4, TRPC5, and TRPC6) in VSMCs and VECs measured by RT-qPCR. (b) Western blots and protein expression levels of TRPCs (TRPC1, TRPC3, TRPC4, TRPC5, and TRPC6) in VSMCs and VECs. *N* = 12. ^*∗*^*p* < 0.05. ^*∗∗*^*p* < 0.01.

RT-qPCR results (Figure 1(a)) showed that mRNA expression levels of TRPC1 and TRPC6 were higher in VECs than VSMCs. By contrast, in VSMCs, mRNA expression levels of TRPC3, TRPC4, and TRPC5 were significantly higher than in VECs. Differences were statistically significant (*p* < 0.05).

We used western blotting to measure protein abundance for five calcium regulatory proteins in VECs and VSMCs and analyzed the results of DAB color development using ImageJ software (Figure 1(b)). After grayscale analysis, we found that TRPC6 (*p* < 0.01) in VECs was significantly more abundant than in VSMCs. In contrast, TRPC4 (*p* < 0.01) and TRPC5 (*p* < 0.05) were more abundant in VSMCs than VECs. The abundance of TRPC1 and TRPC3 differed in the two cell types, but not statistically so (*p* > 0.05).

In summary, we measured the abundance of five calcium regulatory proteins in VECs and VSMCs by qPCR and western blotting. We found that both mRNA and protein levels of TRPC4 and TRPC5 were significantly different in the two cell types. Thus, we choose TRPC4 and TRPC5 for subsequent experiments.

### 3.3. Construction of TRPC4 and TRPC5 Knockdown VSMCs and VECs

**Figure 2:**
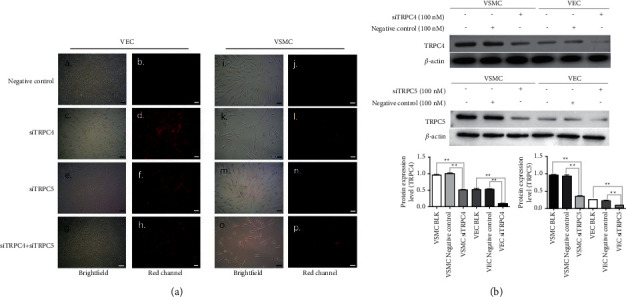
(a) siTRPC4 (100 nM) and siTRPC5 (100 nM) were transfected into VECs and VSMCs, and cells were examined 48 h after transfection by fluorescence microscopy. a and b are unlabeled Cy3 negative controls for transfection of VECs for 48 h; c and d are TRPC4 siRNA (100 nM) for transfection of VECs for 48 h, e and f are TRPC5 siRNA (100 nM) for transfection of VECs for 48 h; g and h are TRPC4 siRNA (100 nM) and TRPC5 siRNA (100 nM) which were cotransfected into VECs for 48 h. Among them, a, c, e, and g are observations of cell morphology after transfection under brightfield, b, d, f, and h are observations of transfection efficiency under the red fluorescence channel, i and j are unlabeled Cy3 negative controls for transfection of VSMCs for 48 h, k and l are TRPC4 siRNA (100 nM) for transfection of VSMCs for 48 h, m and n are TRPC5 siRNA (100 nM) for transfection of VSMCs for 48 h, and o and p are TRPC4 siRNA (100 nM) and TRPC5 siRNA (100 nM) which were cotransfected into VSMCs for 48 h. Among them, i, k, m, and o are observations of cell morphology after transfection under brightfield, and j, l, n, and p are observations of transfection efficiency under the red fluorescence channel. Scale bars = 50 *μ*m. (b) After expressions of TRPC4 and TRPC5 were suppressed using an siRNA strategy, TRPC4 and TRPC5 protein expression levels were measured by western blotting. BLK: blank group. *N* = 12. ^*∗*^*p* < 0.05 vs. VSMC-CTRL, VSMC siTRPC4, and VSMC siTRPC5. ^*∗∗*^*p* < 0.01.

The experimental method used in this study successfully transferred siRNAs into target cells (refer to Supplementary Materials 2 for details). We selected the optimized siTRPC4 and siTRPC5 to transfect VECs and VSMCs (refer to Supplementary Materials 3 for details). The results showed that no red fluorescence was observed in negative controls, but red fluorescence was observed with 100 nM siTRPC4, 100 nM siTRPC5, and both siRNA constructs after transfection for 48 h. The transfection efficiency of TRPC4 and TRPC5 siRNA into VECs and VSMCs is over 90% (Figure 2(a)). Next, we used western blotting to analyze protein expression levels for TRPC4 and TRPC5. The results indicated that transfection of TRPC4 and TRPC5 siRNAs into VECs and VSMCs downregulated the expression of TRPC4 and TRPC5 at protein levels (Figure 2(b)).

### 3.4. Concurrent Knockdown of TRPC4 and TRPC5 Inhibits VSMC Proliferation without Affecting VECs

**Figure 3:**
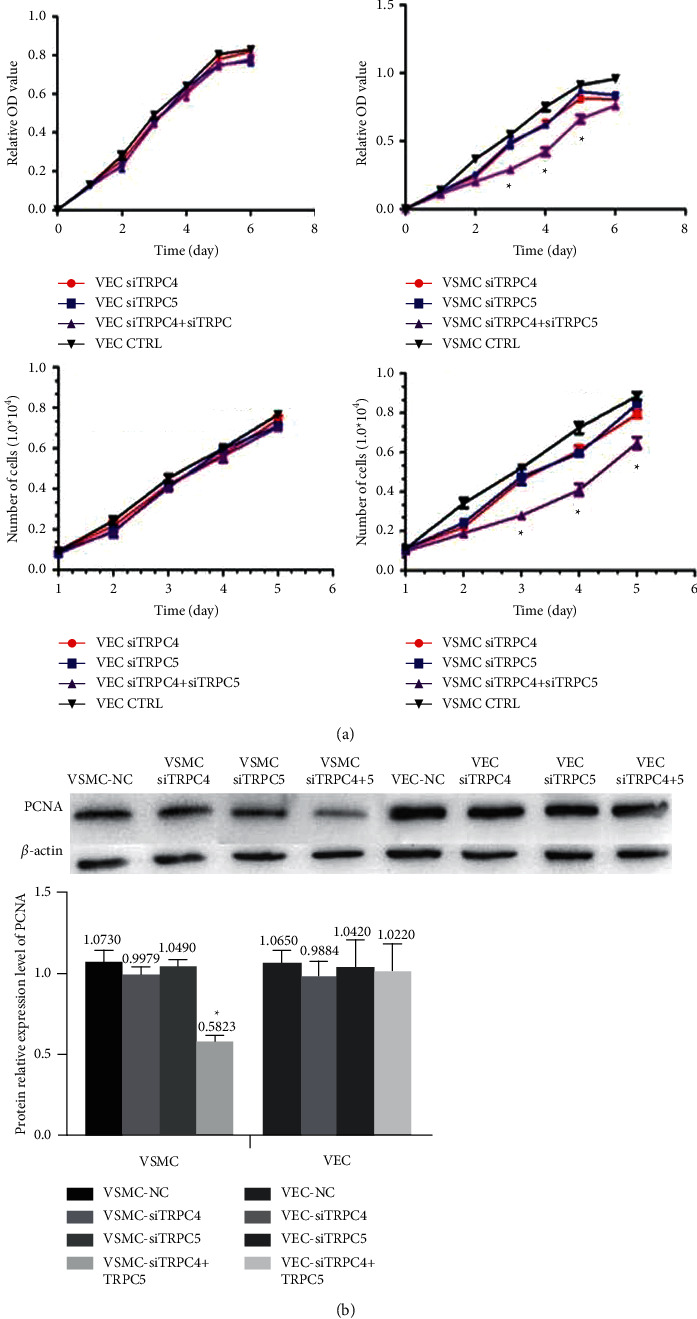
(a) The effects of silencing TRPC4 and TRPC5 on VEC and VSMC proliferation assessed by CCK-8 analysis and viable cell counting. ^*∗*^*p* < 0.05 vs. VSMC-CTRL, VSMC siTRPC4, and VSMC siTRPC5. ^*∗∗*^*p* < 0.01. (b) Western blotting analysis of PCNA protein expression. For both VSMCs and VECs, TRPC4, TRPC5, and combined TRPC4 + 5 interference groups were tested, and the results were compared with the NC group. Data are presented as the means +SEM. *N* = 12. ^*∗*^*p* < 0.05 vs. VSMC-NC.

In this study, we used CCK-8 assays, viable cell counting, and cyclin PCNA detection to examine the effects of TRPC4 and TRPC5 siRNAs on the proliferation of VECs and VSMCs. The results revealed no changes in the proliferative capacity of VECs carrying siRNAs for TRPC4 or TRPC5 alone or in combination. However, in VSMCs, cells cotransfected with TRPC4 and TRPC5 siRNAs displayed more pronounced inhibition of proliferation than cells carrying TRPC4 or TRPC5 siRNA alone (Figure 3(a)). In VSMCs, compared with the scramble siRNA (NC) group, the combined TRPC4 and TRPC5 interference group exhibited downregulated expression of PCNA (*p* < 0.05; Figure 3(b)).

### 3.5. Concurrent Knockdown of TRPC4 and TRPC5 Reduced the Intracellular Calcium Concentration in VSMCs but Not in VECs

**Figure 4:**
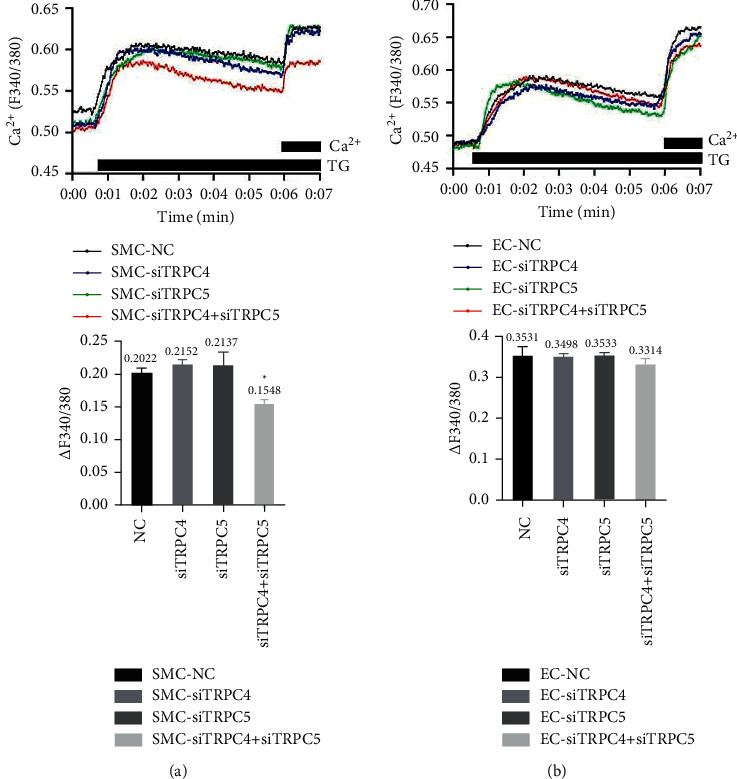
(a) Effects of TRPC knockdown on calcium store depletion in VSMCs. TRPC4, TRPC5, and TRPC4 + TRPC5 were assayed after 7 min. Interfering with TRPC4 and TRPC5 together significantly reduced intracellular calcium levels (*N* = 6, ^*∗*^*p* < 0.05 vs. scramble siRNA (NC)). (b) Effects of TRPC knockdown on calcium store depletion in VECs. TRPC4, TRPC5, and TRPC4 + TRPC5 interference groups were assayed after 7 min. The increase in intracellular calcium ions in each experimental group was essentially restored to the level of the NC group (*N* = 6, ^*∗*^*p* < 0.05 vs. NC).

A 1 *μ*M concentration of thapsigargin (TG) was used to stimulate the depletion of intracellular calcium stores, the external calcium concentration was increased to 2 M, and changes in the intracellular calcium ion concentration were measured. The results showed that, in VSMCs, interfering with TRPC4 or TRPC5 alone did not alter the intracellular calcium ion concentration (*p* > 0.05). However, interfering with both TRPC4 and TRPC5 simultaneously significantly reduced the intracellular calcium concentration (*p* < 0.05; Figure 4(a)). In VECs, interfering with TRPC4 or TRPC5 alone or with both TRPC4 and TRPC5 together did not significantly alter the intracellular calcium ion concentration from the level of the NC group (*p* > 0.05; Figure 4(b)).

## 4. Discussion

The aim of this study was to identify the molecular target that inhibits the proliferation of VSMCs without affecting VECs by studying SOC and related calcium-modulating molecules. We compared expression at mRNA and protein levels in VECs and VSMCs for five Ca^2+^ channel proteins (TRPC1, TRPC3, TRPC4, TRPC5, and TRPC6). Among them, TRPC4 and TRPC5 were significantly differentially expressed in VECs and VSMCs. The expression of TRPC4 and TRPC5 was higher in VSMCs than VECs. The results of this study indicate that, in human coronary artery smooth muscle cells and endothelial cells, downregulation of TRPC4 and TRPC5 can inhibit the proliferation of VSMCs without affecting the proliferation of VECs. When TG was used to deplete the intracellular calcium pool, the intracellular calcium ion concentration decreased significantly. When exogenous calcium ions were added, the intracellular calcium ion concentration recovered. However, in cells in which TRPC4 and TRPC5 were silenced simultaneously, adding exogenous calcium ions did not recover the intracellular calcium ion concentration. Analysis of the calcium concentration showed that cointerference of TRPC4 and TRPC5 significantly affected calcium influx in VSMCs, but not VECs.

The TRPC family comprises seven members (TRPC1-7) that form homo- and heterodimers that regulate intracellular Ca^2+^ concentrations and participate in various physiological and pathological processes [18]. Among them, TRPC4 and TRPC5 belong to the same subfamily and share 69% homology [19]. This study further corroborated the homology of TRPC4 and TRPC5 genes. Our western blotting results showed that protein expression levels for TRPC4 and TRPC5 were almost identical in VECs and VSMCs, and this was further supported by functional experiments. These two genes have a synergistic effect, and silencing both genes inhibited the proliferation of VSMCs to a greater extent than silencing either gene alone. The results confirmed that TRPC4 and TRPC5 are homologous and perform similar functions.

One study showed that TRPC1 protein levels were significantly reduced in aging rat aorta tissue, while TRPC6 was increased dramatically [20]. Further research found that siRNA-mediated suppression of TRPC1 expression can be compensated by TRPC6 upregulation [21]. Another study revealed that TRPC5 can interact with molecules such as TRPC4 and TRPC6, thereby regulating their physiological functions [22]. TRPC has a tendency to form heteropolymers, and the loss or downregulation of one or more TRPC proteins may be compensated by other TRPCs [23]. Whether this mechanism also exists between TRPC4 and TRPC5 requires further research.

Various pathophysiological states are associated with increased and decreased TRPC channel expression and abnormal channel opening and closing activity [24]. Recent studies have shown that TRPC4 participates in pulmonary hypertension by affecting the proliferation of endothelial cells and smooth muscle cells [25]. The use of TRPC inhibitors and specific TRPC channel blockers can reduce the Ca^2+^ concentration and regulate cell proliferation [26]. In our study, TRPC4 and TRPC5 expression was detected at both mRNA and protein levels in both VECs and VSMCs. However, expression levels were higher in VSMCs than VECs. Subsequent experiments showed that the proliferation of VSMCs was significantly decreased following silencing of TRPC4 and TRPC5. In contrast, proliferation of VECs was not significantly affected by silencing of these genes. Our study is that the expression of TRPC channels has changed, which affects the physiological state of VSMCs and VECs. The mechanism of downregulating TRPC4 and TRPC5 to inhibit VSMCs' proliferation is achieved by inhibiting the decrease in SOCE mediated by TRPC4 and TRPC5, resulting in a decrease in cellular calcium influx. We need to further establish atherosclerosis animal models to determine the effect of TRPC4 and TRPC5 on the proliferation of VSMCs and VECs under pathological conditions. There is a study showing that, in endothelial cells, such as TRPC1, TRPC4 also promotes capacitive calcium influx [27]. Whether this capacitive calcium influx is related to SOCE is unclear, but our experiments show that downregulation of TRPC4 and TRPC5 has no effect on the proliferation of endothelial cells, so we can speculate whether capacitive calcium influx supplements the function of TRPC4, and further exploration is needed. Uncontrolled cell proliferation may be associated with high levels of apoptosis [28]. Furuya et al. also showed that an increase in intracellular free calcium ions can lead to cell apoptosis [29]. Therefore, there is a close relationship between apoptosis and cell proliferation. In our study, apoptosis was not detected, and this will be further explored in subsequent experiments.

In conclusion, the present study clearly demonstrated that downregulation of TRPC4 and TRPC5 can inhibit smooth muscle cell proliferation, which provides potential drug targets for establishing new drug-eluting stents.

## Figures and Tables

**Table 1:** Primer sequences used in the study.

Gene	Forward	Reverse
TRPC1	5′-TGCGTAGATGTGCTTGGGAG-3′	5′-TCGATTGCCACCAAAAGTGC-3′
TRPC3	5′-ACCAAGGTCAGGAAGTGCAAA-3′	5′-ATCACTGTCATGCGTCTCAGG-3′
TRPC4	5′-GAGGCGAGCTGCTGATAACT-3′	5′-ATCCCAGGACTTCAAAGCGG-3′
TRPC5	5′-TGAACTCCCTCTACCTGGCA-3′	5′-GGTCCTAAGTGGGAGTTGGC-3′
TRPC6	5′-CAAGTCCGGCTTACCTGTCA-3′	5′-TCACCACTCTGGAGCGTTTC-3′
*β*-Actin	5′GTTAAGTGGGATCGAGACATGTAAG-3′	5′-ATTCATCCAATCCAAATGCGGC-3′

## Data Availability

All the data generated or analyzed during this study are included within this published article as well as its supplementary information files.

## Supplementary Materials

Supplementary Materials Morphological comparison of vascular smooth muscle cells and vascular endothelial cells, siRNA transfection into VECs and VSMCs, and construction of TRPC4 and TRPC5 knockdown vascular smooth muscle cells and vascular endothelial cells are presented.
