# Supplementary material for: Downregulation of TRPC4 and TRPC5 Inhibits Smooth Muscle Cell Proliferation without Affecting Endothelial Cell Proliferation
Source: Genet Res (Camb). 2021 Nov 27;2021:2949986. doi: 10.1155/2021/2949986 (PMC8643255; doi:10.1155/2021/2949986)
Supplement: Supplementary Materials — Morphological comparison of vascular smooth muscle cells and vascular endothelial cells, siRNA transfection into VECs and VSMCs, and construction of TRPC4 and TRPC5 knockdown vascular smooth muscle cells and vascular endothelial cells are presented. [file 2949986.f1.zip › 2949986.f1/Supplementary Materials(1).docx]

**Supplementary materials 1**

Smooth muscle cells had a great of different growth patterns at different densities. They exhibited a polygonal or triangular shape at low densities, with good cell refraction and clear cell outline. When the confluence of SMC cells reaches 50% to 60%, SMC cells showed a typical fusiform structure growth, which was more uniform and the spindle structure was more typical, the refractive index is good, and the cell outline was clear. When the SMC confluence reached 100%, the cells showed a very typical long spindle shape, which was a fusiform shape with a confluence of 50% to 60%, and the length of SMC cells was significantly increased. The arrangement rules are neat, the refractive index is good, and the cell boundaries are relatively clear.

Endothelial cells had slightly different growth patterns at different densities. The EC cells exhibited a polygonal shape at low cell densities, and there were elongated pseudopodia connections between cells, and the cells had good refractive index and clear cell contours. The cell exhibited an atypical fusiform structure, the cell refractive index is good, and the cell outline is relatively clear when the it grows to a confluence of 50% to 60%. The cell did not exhibit a typical fusiform structure but is rhomboid-like when it grows to a confluence of 100%, with a slightly lower refractive index and an unclear cell outline.


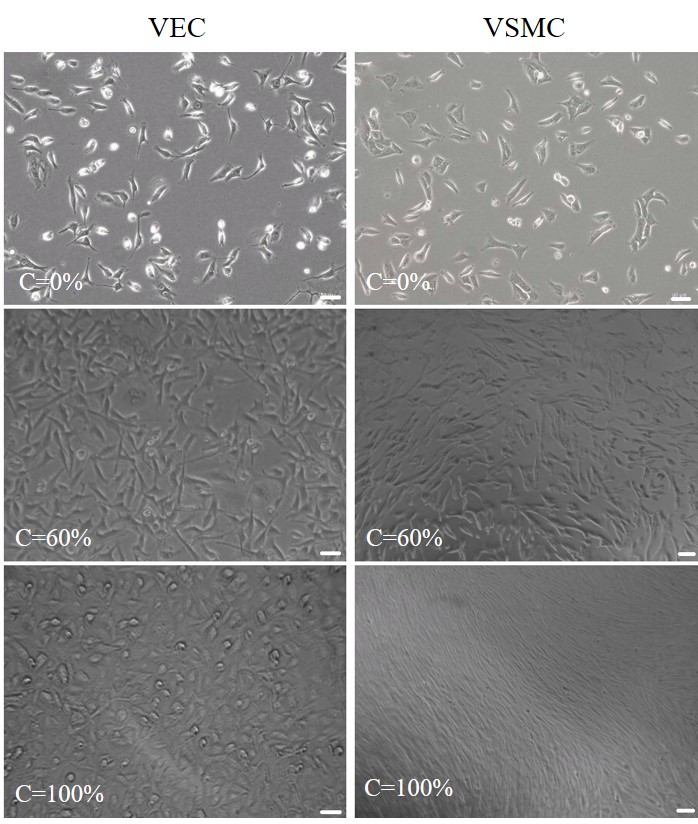


**Supplementary figure 1.** Morphological comparison of vascular smooth muscle cells and vascular endothelial cells. C represents confluence. All cells are passage 3. All scale bar are 50 μm.

**Supplementary materials 2**

Using fluorescence microscopy, we observed negative control VECs not transfected with cy3 label and cy3-labelled positive control VECs for 48 h. Negative control VMSCs not transfected with cy3 label and cy3-labelled positive control VMSCs were also observed for 48 h. The results showed that the experimental method used in this study successfully transferred siRNAs into target cells and caused no significant changes in cell morphology, indicating that siRNAs were compatible with the staining system.


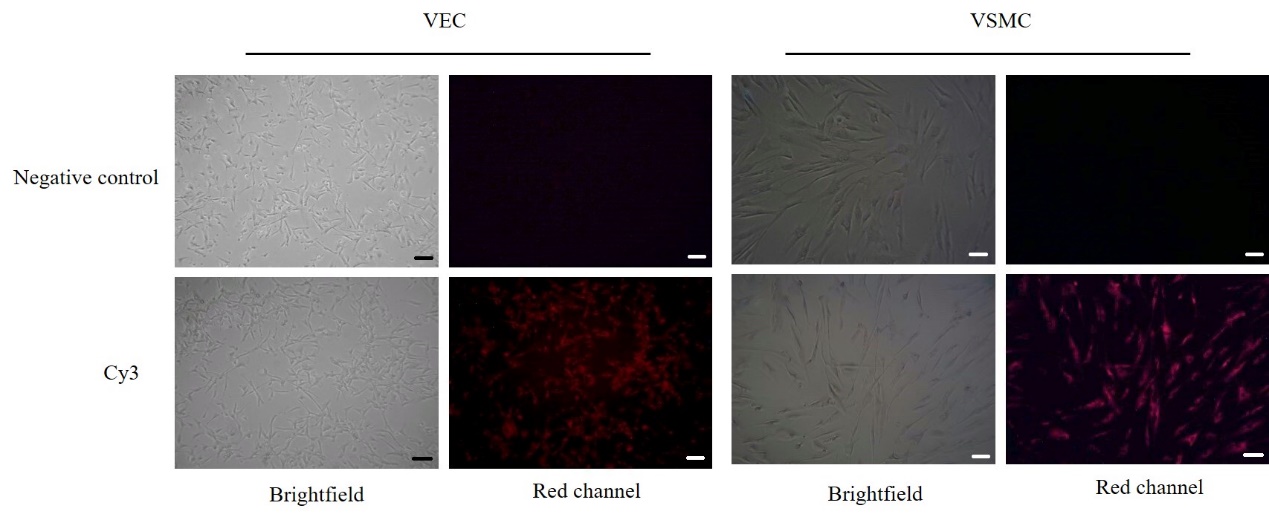


**Supplementary figure 2.** The siRNA transfection into VECs and VSMCs. All scale bars are 50 μm.

**Supplementary materials 3**

From the preliminary experiment, the optimized siTRPC4 sequence is group (2): 5’-GGCCTAAATCAATTGTACT-3’, the optimized siTRPC5 sequence is group (1): 5’-GACACGAATTCACCGAGTT-3’


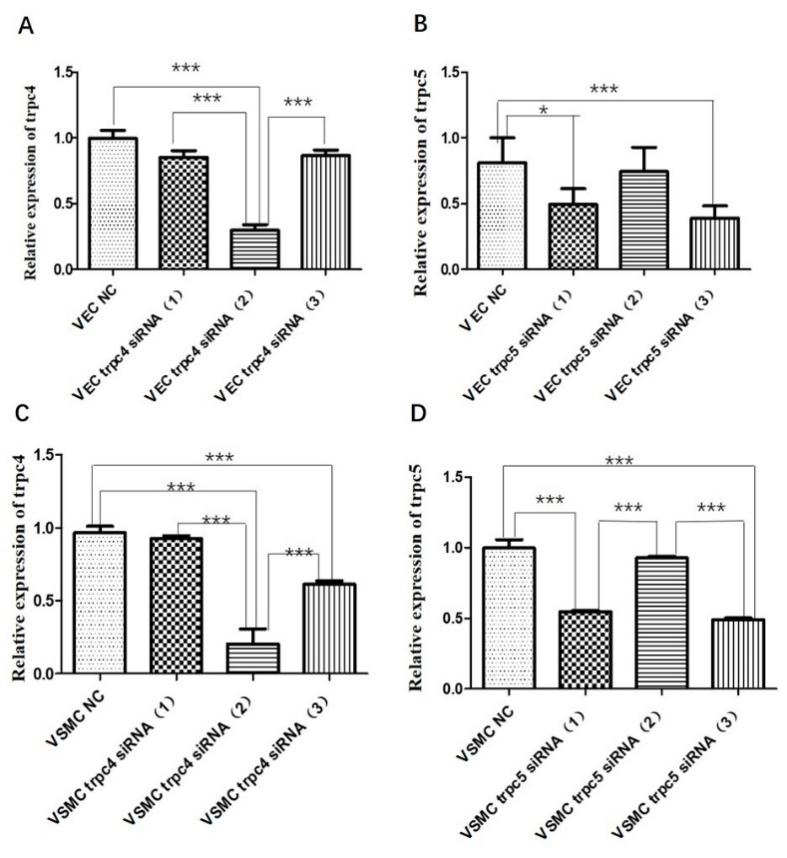


**Supplementary figure** **3.** (A) Silencing of TRPC4 transcription in VECs using TRPC4 siRNA (1), (2) and (3). (B) Silencing of TRPC5 transcription in VECs using TRPC5 siRNA (1), (2) and (3). (C) Silencing of TRPC4 transcription in VSMCs using TRPC4 siRNA (1), (2) and (3). (D) Silencing of TRPC5 transcription in VSMCs using TRPC5 siRNA (1), (2) and (3). Results are expressed as the mean ± SD of three experiments (***p <0.01).
