# Supplementary material for: Downregulation of TRPC4 and TRPC5 Inhibits Smooth Muscle Cell Proliferation without Affecting Endothelial Cell Proliferation
Source: Genet Res (Camb). 2021 Nov 27;2021:2949986. doi: 10.1155/2021/2949986 (PMC8643255; doi:10.1155/2021/2949986)
Supplement: Supplementary Materials — Morphological comparison of vascular smooth muscle cells and vascular endothelial cells, siRNA transfection into VECs and VSMCs, and construction of TRPC4 and TRPC5 knockdown vascular smooth muscle cells and vascular endothelial cells are presented. [file 2949986.f1.zip › 2949986.f1/Supplementary materials.docx]

**Supplementary materials**

Morphological comparison of vascular smooth muscle cells and vascular endothelial cells, siRNA transfection into VECs and VSMCs, construction of TRPC4 and TRPC5 knockdown vascular smooth muscle cells and vascular endothelial cells were presented in supplementary materials.

**Supplementary figure 1.** Morphological comparison of vascular smooth muscle cells and vascular endothelial cells. C represents confluence. All cells are passage 3. All scale bar are 50 μm.

**Supplementary materials 2**

Using fluorescence microscopy, we observed negative control VECs not transfected with cy3 label and cy3-labelled positive control VECs for 48 h. Negative control VMSCs not transfected with cy3 label and cy3-labelled positive control VMSCs were also observed for 48 h. **Supplementary figure 2.** The siRNA transfection into VECs and VSMCs. All scale bars are 50 μm.

**Supplementary materials 3**

From the preliminary experiment, the optimized siTRPC4 sequence is group (2): 5’-GGCCTAAATCAATTGTACT-3’, the optimized siTRPC5 sequence is group (1): 5’-GACACGAATTCACCGAGTT-3’

**Supplementary figure** **3.** (A) Silencing of TRPC4 transcription in VECs using TRPC4 siRNA (1), (2) and (3). (B) Silencing of TRPC5 transcription in VECs using TRPC5 siRNA (1), (2) and (3). (C) Silencing of TRPC4 transcription in VSMCs using TRPC4 siRNA (1), (2) and (3). (D) Silencing of TRPC5 transcription in VSMCs using TRPC5 siRNA (1), (2) and (3). Results are expressed as the mean ± SD of three experiments (***p <0.01).
